# Detection of Five Mycotoxins in Different Food Matrices in the Malaysian Market by Using Validated Liquid Chromatography Electrospray Ionization Triple Quadrupole Mass Spectrometry

**DOI:** 10.3390/toxins11040196

**Published:** 2019-03-31

**Authors:** Ali Mohamed Ali Alsharif, Yeun-Mun Choo, Guan-Huat Tan

**Affiliations:** 1 Department of Chemistry, Faculty of Science, University of Mala, Kuala Lumpur 50603, Malaysia; alshariiiif@yahoo.com; 2 Arab Centre for Desertification and Development of Saharian Societies, Murzuk, Libya

**Keywords:** mycotoxins, aflatoxin, ochratoxin, Plackett–Burman design, Box–Behnken design, QuEChERS, LC-MS/MS

## Abstract

Mycotoxins are common food contaminants which cause poisoning and severe health risks to humans and animals. The present study applied chemometric approach in liquid chromatography-tandem mass spectrometry (LC-MS/MS) optimization for simultaneous determination of mycotoxins, i.e., aflatoxins B1, B2, G1, and G2, and ochratoxin A. The validated quick, easy, cheap, effective, rugged, and safe (QuEChERS)-LC-MS/MS method was used to study the occurrence of mycotoxins in 120 food matrices. The recovery ranges from 81.94% to 101.67% with relative standard deviation (RSD) lesser than 11%. Through the developed method, aflatoxins were detected in raisin, pistachio, peanut, wheat flour, spice, and chili samples with concentration ranges from 0.45 to 16.93 µg/kg. Trace concentration of ochratoxin A was found in wheat flour and peanut samples which ranged from 1.2 to 3.53 µg/kg. Some of the tested food samples contained mycotoxins of above the European legal maximum limit.

## 1. Introduction

Aflatoxins and ochratoxins are mycotoxins produced by *Penicillium* and *Aspergillus* species and are known to contaminate food, feed, and other raw materials. Mycotoxin poisoning in food and feed is a high-risk health problem in humans and animals [1]. Aflatoxin B1, B2, G1, and G2 (AFB1, AFB2, AFG1, and AFG2) are the most common aflatoxins found in food and cause severe health risk when ingested in contaminated food. AFB1 is known to be a potent cancer-causing agent in both humans and animals [2]. Ochratoxin A (OTA) is listed in the International Agency for Research on Cancer (IARC) Group 2B carcinogens list [3]. Consumers are affected by mycotoxins through the consumption of contaminated food, such as fresh and dried fruits, cereals, nuts, fruit juice, processed cereal products, meat, milk, and eggs [4]. The mycotoxin content in food depends on the fungi type, commodity composition, harvesting conditions, food handling and storage, and other factors, such as moisture, relative humidity, temperature and mechanical damage, oxygen, carbon dioxide, substrate composition, insects, and spore load [5]. Various national and international organizations have established maximum level of mycotoxin content in food and feed, to safeguard food quality and consumers health [6].

Liquid chromatography-tandem mass spectrometry (LC-MS/MS) is extensively applied for quantitative and qualitative mycotoxin analyses in different food matrices [7,8,9,10]. Electrospray ionization (ESI) is the most popular method for ionization of molecules in liquid form and is compatible with most chromatographic separation systems. The effectiveness of ionization depends on many factors, such as analyte type, applied voltage, solvent system, drying gas parameters, and eluent flow rate. Developing and validating a quantitative/qualitative in LC-MS/MS system to achieve the optimum sensitivity and selectivity is often time-consuming and complicated [11]. Therefore, it is important to optimize factors that influence retention time and peak area to improve the separation efficiency [12]. In LC-MS/MS, system development and optimization is usually carried out using conventional univariate method [13]. In this approach, the effect of one parameter is investigated by keeping the other factors constant and changing the value of a single variable one at a time. The univariate optimization procedure is time-consuming [14], unable to evaluate more than one variable at a time (not economical) [15], and interactions among factors that may affect the ion source are not evaluated [16]. These difficulties can be addressed by using a multivariate optimization procedure [17]. Multivariate optimization method investigates the interaction between experimental variables efficiently and has been successfully applied for the optimization of liquid chromatographic tandem mass spectrometry analyses [18,19,20,21].

Many sample pre-treatment methods, such as liquid–liquid extraction, solid–liquid extraction, and solid phase extraction (SPE) developed for mycotoxin analysis in food samples aims at reducing the matric effects. The quick, easy, cheap, effective, rugged, and safe (QuEChERS) technique, with or without dispersive SPE, is one of the most widely used sample pre-treatment method for mycotoxin analysis and other contaminants in food samples [22,23,24,25].

The present study aims at applying the chemometric approach for the optimization of the LC-MS/MS method to make the ionization more efficient with maximum peak area and shorter analysis time (retention time). The chemometric approach was carried out by using Plackett–Burman design (PBD) in the screening step to determine significant factors. Meanwhile, the Box–Behnken design (BBD) was applied for the optimization of significant factors determined by PBD method. The LC-MS/MS chemometric optimization approach has better advantages as compared to the time consuming conventional univariate optimization method and served as an attractive alternative approach. The LC-MS/MS system was combined with the QuEChERS-dispersive SPE (high-, non-, and low-fat sample) techniques to determine the natural occurrence of multi-mycotoxins (AFB1, AFB2, AFG1, AFG2, and OTA) in various commercial food samples. All samples were purchased from Kajang and Kuala Lumpur, Malaysia. The determination of mycotoxin contents enabled a clearer vision of mycotoxin occurrence in various food matrices and production quality.

## 2. Results

### 2.1. Optimization of the MS/MS Conditions

The five mycotoxins (AFB1, AFB2, AFG1, AFG2, and OTA) were directly injected into the mass system and analyzed under a mass full scan mode. In LC-MS/MS, precursor ions selected in the first quadrupole was fragmented in the second quadrupole (collision cell) to produce an analyte-specific product ion and was subsequently monitored in multiple reaction monitoring (MRM) data acquisition mode in the third quadrupole. In this study, a full-scan mode (100–500 *m/z*) was applied for the LC-MS analysis of mycotoxins. The results revealed that all mycotoxins could produce precursor ion [M + H]^+^ under ESI-positive mode. The selection of product ions was carried out by varying the collision energy to yield the best intensity. The two highest abundant fragments were selected for subsequent experiments. Table 1 summarizes the mycotoxins MS data. 

### 2.2. Univariate Optimization

The mobile phase additives and their effects on the peak area in this optimization study were formic acid (A), acetic acid (B), ammonium acetate (C), ammonium formate (D), ammonium formate/formic acid (AC), ammonium formate/acetic acid (BC), ammonium acetate/formic acid (AD), and ammonium acetate/acetic acid (BD). The results indicated that the combination of ammonium format/formic acid in the mobile phase gave the best chromatographic results and was selected as additives for the mobile phase A (H_2_O). The conventional univariate method was used in primary study to evaluate levels of parameters. In this method, value of one-factor was changed, and others were kept constant. Eleven factors were optimized under the univariate method, i.e., pH value, % of organic solvent, flow rate, column temperature, injection volume, sheath gas flow rate, sheath gas temperature, gas flow rate, gas temperature, and nebulizer pressure and the optimized values are shown in Table 2. 

### 2.3. Plackett–Burman Design (PBD)

Plackett–Burman design provides information of each factor with a minimum number of the experiment [26]. In this study, PBD optimization was carried out to evaluate 11 factors and determine which factors have a significant effect on the total chromatographic peak area (TCPA) and mean retention time (MRT) [27,28] on the mycotoxins (AFB1, AFB2, AFG1, AFG2, and OTA). All experiments were carried out by using mycotoxins mixture which contained 100 µg/L each of AFB1, AFG1, and OTA; and 30 µg/L each of AFB2 and AFG2 in a total of 24 experiments. The factors and their respective upper and lower levels were pre-selected using the univariate method which is within the instrument operation parameters recommended by the manufacturer (Table 2).

**Figure 1:**
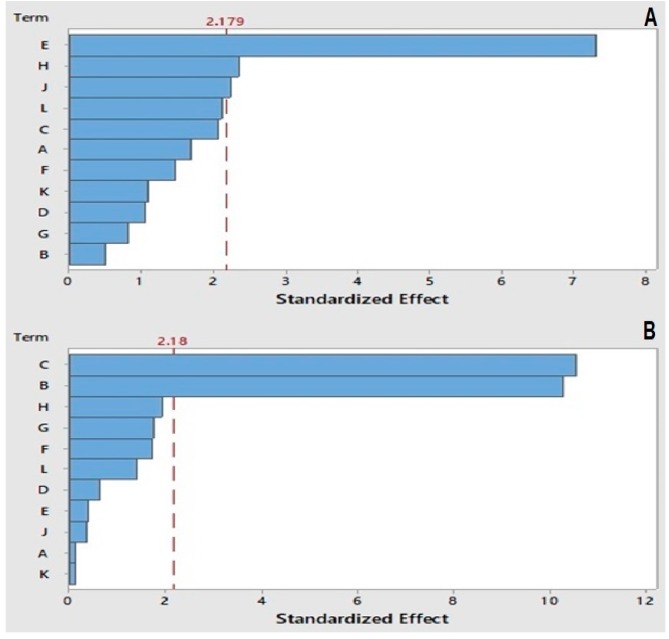
Plackett–Burman design (PBD) standardized Pareto chart for (**A**) total chromatographic peak area (TCPA) and (**B**) mean retention time (MRT).

The analysis of variance (ANOVA) was used to assess model adequacy and significant variables were identified by using F-test [29]. The polynomial fit quality was expressed by the coefficient [30], in which R^2^ = 1 is the best quality and the lowest value for R^2^ is set to be higher than 0.8 [31]. In this study, experimental R^2^ and adjusted R^2^ were determined as 90.33% and 85.17%, respectively for TCPA, and 95.03% and 90.47%, respectively for MRT. The Pareto chart plot (Figure 1) indicates that TCPA was significantly affected by the sample injection volume, gas flow, and gas temperature. Meanwhile, MRT was significantly influenced by the mobile phase flow rate and organic solvent percentage. Other factors did not have any significant effects on TCPA or MRT.

### 2.4. Box–Behnken Design (BBD)

The five significant factors obtained from PBD optimization, i.e., % of organic solvent, flow rate, injection volume, gas flow, and gas temperature were subjected to further optimization by using BBD method. Other parameters, such as the pH value, column temperature, sheath gas flow, sheath gas temperature, and nebulizer pressure were set at pH 3, 30 °C, 11 L/min, 250 °C, 20 psi, respectively, which was determined in the earlier univariate study. The variables used in the generation of BBD experimental levels are summarized in Table 2.

The analysis of variance (ANOVA) by using the quadratic model indicated that the good-fit for TCPA and MRT with *p* > 0.05 (lack-of-fit values of 0.593 and 0.825, respectively) as in Table 3. The experimental R^2^ and adjusted R^2^ values were 98.92% and 97.83%, respectively for TCPA; and 99.15% and 98.41%, respectively for MRT. The high R^2^ values indicated goodness-of-fit and a correlation between the observed and predicted values. 

**Figure 2:**
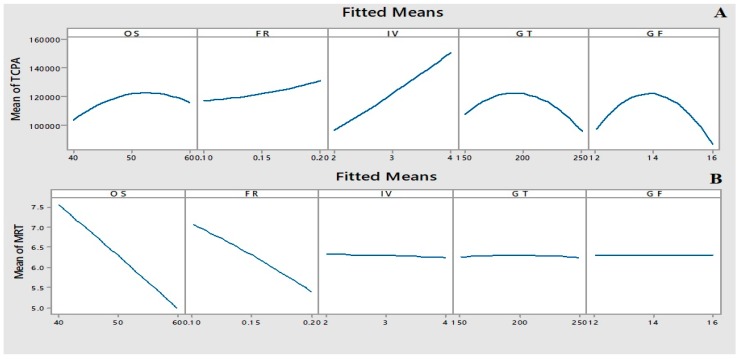
BBD main effects Plots for (**A**) TCPA and (**B**) MRT.

The main effect plot (Figure 2) shows that injection volume and TCPA increased hand-in-hand. However, increases in gas flow or temperature caused TCPA to increase initially, but then subsequently decreased. In the case of MRT response, increasing the organic solvent percentage and flow rate of mobile phase caused reduction in retention time, while other factors, such as injection volume, gas flow, and temperature had no significant effect on MRT. The relation between responses (TCPA and MRT) and instrumental parameters are shown in the response surface plot (Figure S1). Hence, the final optimized parameters from BBD study were organic solvent = 60%, flow rate = 0.2 mL/min, injection volume = 4 µL, gas flow = 14 L/min, and gas temperature = 170 °C. Table 2 shows the final optimized parameters for LC-MS/MS using combined univariate and multivariate approach. 

### 2.5. Method Performance

Method performance was carried out in two steps. First, the performance of LC-MS/MS was evaluated for linearity (regression equation, correlation of determination R^2^), precision (intra- and inter-day), and instrument detection limit (IDL), and the parameters are summarized in Table 4. Good linearity values were obtained for all five mycotoxins spiked in MeOH and aflatoxin-free peanut extract. The R^2^ was determined as greater than 0.9992 with a concentration range of 0.012 to 50 µg/L. Satisfactory precision was obtained with RSD lower than 20% in both mycotoxins spiked-MeOH and aflatoxin-free peanut extract samples. The intra-assay precision (repeatability) was assessed by continuous analysis on ten replicates of mycotoxins mixtures (10 µg/L of each AFB1, AFG1, and OTA; and 3 µg/L of each AFB2 and AFG2) in a single day. The inter-day precision (40 replicates) was estimated based on 10 runs per-day (10 replicates) for four days. The experiments provided acceptable repeatability results with RSD of lesser than 4%. To evaluate the IDL, mycotoxins mixture was analyzed in eight replicates, and the results indicated that the IDL values in the range of 1.41 to 3.61 ng. Figure S2 shows the LC-MS/MS total ion chromatogram (TIC) and MRM chromatogram of the mycotoxin mixture obtained under BBD.

In the second step, the QuEChERS-LC-MS/MS s was validated. The method performance was estimated from the matrix-matched calibration curve (to provide an accurate quantification of different varieties) [32]. The linearity with each calibration curve was constructed using individual mycotoxin average peak area against a corresponding concentration. The sensitivity of the method was expressed as LOD and LOQ. The method showed good linearity with a correlation coefficient of greater than 0.9967 for all mycotoxins spiked in the seven levels for each analyzed matrix. The method demonstrated good sensitivity with LOD that ranged from 0.05 to 0.1 µg/L or µg/kg, and LOQ that ranged from 0.08 to 0.3 µg/L or µg/kg, within the accepted S/N ratio of 3:1 and 10:1 for LOD and LOQ, respectively, which is much lower than the allowable maximum limit for both aflatoxins and OTA permissible under the European Commission. The linearity and sensitivity results are summarized in Table 5.

The recovery was determined at different concentrations spiked in selected food matrices, and the results are summarized in Table 6. The intra-day and inter-day precision was determined at different concentrations and expressed as the relative standard deviation (RSD). The intra-day precision (triplicates) was estimated by performing three extractions per day. The inter-day precision (12 replicates) was investigated based on three extractions per day (triplicates) for four days and reported as the relative standard deviation (RSD). The results indicated good intra-day and inter-day precision below the acceptance limit of the method (lesser than 20%), with intra-day and inter-day precision ranges from 0.12 to 7.25% and 0.23 to 10.28%, respectively. The recovery of the target analytes ranges from 81.94 to 101.67% in different food sample matrices and was within the recommended range for validation method of aflatoxins and OTA (70–110%). The overall method of performance has satisfied the requirements established by the European Union (EU) legislation [33]. The performance results revealed the suitability of the validated method for the determination of trace concentration of mycotoxins (aflatoxins and ochratoxins). Matrix effect calculation and selectivity were not taken into consideration in this study as the matrix-matched calibration curve applied in the study is able to eliminates or reduced the matrix effect [34,35]. 

### 2.6. Comparison of the Developed Method with Other Methods

The efficiency of the optimized LC-MS/MS method using chemometric approach combined to QuEChERS was compared to other QuEChERS methods for the determination of multi-mycotoxins from different food samples (Table 7). The present method revealed excellent overall results for the simultaneous separation and determination of mycotoxins (AFB1, AFB2, AFG1, AFG2, and OTA). The LOQs of the present method was lowest among all the compared methods. The recovery and precision were better or comparable with other methods. 

The data also highlighted the advantages of the using combined univariate-multivariate approach over conventional optimization method, as the multivariate approach takes interaction effects into consideration. The combined univariate-multivariate approach requires shorter optimization steps and time, lesser cost, better method performance, and higher accuracy, and most suitable for optimization of multi-parameters methods such as LC-MS/MS. It is notable to add that the developed method can be applied for routine analysis of multi-mycotoxins in the studied matrices.

### 2.7. Occurrence of Studied Mycotoxins in Real Food Samples

Mycotoxins (AFB1, AFB2, AFG1, AFG2, and OTA) were analyzed in 120 food samples, and the results are summarized in Table 8. The food samples consisted of non- and low-fat samples (apple, grape, orange, and pomegranate juices; wheat and barley flour; dried figs, raisins, chili powder, and spices) and a high-fat sample (non-roasted peanut and roasted pistachio). These samples were purchased from Kajang and Kuala Lumpur, Malaysia. Each sample was prepared in triplicate for extraction and LC-MS/MS analysis under optimized conditions. Aflatoxins were detected in 19 out of the 120 tested food samples with concentration ranges from 0.45 to 16.93 µg/kg. Aflatoxins were detected in the range of 0.76 to 10.23 µg/kg in the two of the non-roasted peanut samples, and 0.81 to 10.15 µg/kg in four roasted pistachio samples. All aflatoxins-positive pistachio samples exceeded the legal limit for nuts (2 µg/kg of AFB1) for direct human consumption or use as food ingredients [39]. The aflatoxins contamination results were in agreement with previously reported studies on aflatoxin contamination in Malaysia peanuts [40,41,42]. This observation may be attributed to the bad harvesting or storage conditions, especially in the tropical climates and the storage conditions in Malaysia [43,44]. The occurrence of aflatoxins in dried fruits group were found only in raisin samples, with all dried fig samples aflatoxins-free. The dried raisins results revealed 2 out of 10 raisin sample were contaminated with all four type of aflatoxins (AFB1, AFB2, AFG1, and AFG2), ranging from 0.84 to 5.67 µg/kg, and exceeded the maximum limit set by EU regulations for direct human consumption (2 µg/kg of AFB1) [45] in both samples.

Aflatoxins (AFB1, AFB2, AFG1, and AFG2) were detected in three of flour samples in the range of 0.45 to 10.12 µg/kg, while the barley flour was free from aflatoxins. Four chilli and four mixed spices were positive for aflatoxins (contaminated with AFB1 and other aflatoxins, such as AFB2, AFG1, and AFG2). The number of samples contaminated with AFB1 or sum of aflatoxins found to have exceeded the maximum limit set by EU regulations are two wheat flour (2.0 µg/kg for AFB1 and sum of aflatoxins 4.0 µg/kg) [39], and three each of chilli and mixed spices (5.0 µg/kg for AFB1 and sum of aflatoxins 10.0 µg/kg) [39] (Table 8).

In addition to aflatoxins, wheat flour and peanut samples showed the presence of trace amount of ochratoxin A (OTA). The concentrations of OTA in wheat flour and peanut sample were 1.2 µg/kg and 1.20 to 3.53 µg/kg, respectively. The detected amount of OTA was below the international, and European legal maximum limit for OTA in cereal products [39]. Mycotoxins were not detected in fruit juice and fig samples. It is common to detect aflatoxins above the European legal maximum limit in raisin, non-roasted peanut, wheat flour, chilli, and spices [46]. Therefore, it is especially important to follow the recommendations and guidelines to reduce the production of aflatoxins in food by taking extra care in the food production steps (from cultivation process to consumers).

## 3. Conclusions

The occurrence of five mycotoxins (AFB1, AFB2, AFG1, AFG2, and OTA) in 120 commercial food samples from two regions in Malaysia (Kuala Lumpur and Kajang) was evaluated by using LC chromatography-tandem mass spectrometry technique. The detection of mycotoxins using LC-MS/MS was optimize utilizing two strategies, i.e., univariate and multivariate optimization methods. The multivariate optimization procedure consisted of the screening of 11 factors using Plackett–Burman design (PBD). The significant factors obtained from PBD were then subjected to further optimization by using Box–Behnken design (BBD). Method performance was carried out for LC- MS/MS and QuEChERS-LC-MS/MS, which revealed good instrument performance with overall better results (lower LODs; better recovery and precision) when compared with other QuEChERS-LC-MS/MS methods. The validated LC-MS/MS method combined with QuEChERS method was applied for the separation and determination of five mycotoxins in various commercial food. Mycotoxins were detected in a number of foods in the range of 0.45 to 16.93 µg/kg. Although the types of food samples examined was limited, the results gave an initial overview on the food quality from two regions in Malaysia. In some tested food, the mycotoxins content exceeded the EU limit which may be caused by inadequate harvesting and storage conditions. The present study demonstrated that the multivariate method of using PBD/BBD coupled with response surface method is a legitimate alternative for the univariate method in the optimization of LC-MS/MS for simultaneous determination and separation of mycotoxins. 

## 4. Materials and Methods 

### 4.1. General

Aflatoxins with standard mixture of AFB1 (1 mg/L), AFG1 (1 mg/L), AFB2 (0.3 mg/L), and AFG2 (0.3 mg/L) in MeOH, and Ochratoxin (OTA) standard (50 mg/L), glacial acetic acid, and formic acid were obtained from Sigma-Aldrich (Darmstadt, Germany). Ammonium formate and ammonium acetate were obtained from Agilent Technologies (Santa Clara, CA, USA). Sodium chloride and anhydrous magnesium sulfate, primary secondary amine (PSA), C18 sorbent, and LCMS-grade acetonitrile and methanol were purchased from Merck (Darmstadt, Germany). Ultra-pure water (ELGA) was used throughout this study. 

### 4.2. Samples

120 food samples were analyzed for mycotoxin content. Non- and low-fat samples (apple, grape, orange, and pomegranate juices; wheat and barley flour; dried figs, raisins, chili powder, and spices) and a high-fat sample (non-roasted peanut and roasted pistachio) were purchased from Kajang and Kuala Lumpur, Malaysia. The samples were stored in a dark cold place at temperature of below 5 °C. The dried fruits and nuts were pulverized into fine homogeneous granules by using an electric spice and nut grinder. Then the homogenized sample was kept in a tightly closed vial and stored at 4 °C until analysis. In the performance study, the samples were spiked with three appropriate levels (Table 6) of mycotoxins and kept in the laboratory to evaporate the solvent from non-liquid samples. 

### 4.3. LC-MS Instrumentation

LC-MS/MS was obtained from Agilent 6490 QQQ (Agilent Technologies, Santa Clara, CA, USA) mass-spectrometer equipped with Agilent 1290 series Rapid Resolution LC system with Agilent Jet-Stream ESI interface (Agilent Technologies, Santa Clara, CA, USA). Mycotoxin separation was achieved on a reversed phase C18 column (ODS) (150 mm × 2.1 mm; 5 µm). Data acquisition, processing, and instrument control were performed through the Mass Hunter Workstation B.06.01 software. MS parameters were optimized by using mycotoxin mixture that contained 100 µg/L AFB1, AFG1, and OTA standards, and 30 µg/L of AFB2 and AFG2 standards in MeOH.

### 4.4. Non- and Low-Fat Sample Preparation

The extraction of mycotoxins from fruit juices, dried figs, dried raisins, wheat flour, barley flour, chilli, and spices were performed according to the QuEChERS method [47]. In total, 2.0 g of homogeneous solid food (or 2 mL liquid sample) was weighed and transferred to a 50 mL polypropylene centrifuge tube. Then, 10 mL of acetonitrile acidified with 1% acetic acid and 7.5 mL of cold water was added to the tube, shaken for 1 min, and vortexed for 4 min. Then, 4 g of anhydrous magnesium sulphate and 1 g of sodium chloride was added to the mixture and shaken for 3 min, and then centrifuged for 6 min at 7500 rpm. Next, 4 mL from the upper organic phase was pipetted out and added to 15 mL centrifuge tube containing 0.2 g PSA and 0.6 g of fine powder anhydrous magnesium sulphate. The extract was further shaken for 2 min and centrifuged at 4000 rpm for 5 min. Then, 2.5 mL of the extract was evaporated to dryness by a rotary evaporator and reconstituted with 1 mL of methanol and filtered through a 0.22 μm nylon syringe filter prior to the LC-MS/MS analysis.

### 4.5. High-Fat Sample Preparation 

The extraction of mycotoxins from nuts sample were performed according to the QuEChERS method [37]. A total of 2.5 g of the homogeneous nut samples was weighed and transferred to a 50 mL polypropylene centrifuge tube. Then, 20 mL acetonitrile aqueous solution (80:20, *v*/*v*) that contained 0.1% formic acid was added to the mixture and shaken for 30 min at 300 rpm. The mixture was then centrifuged for 5 min at 8000 rpm and the supernatant was transferred into a clean vial. The extraction process was repeated twice. Next, 4 g of magnesium sulfate, 1 g of sodium chloride, 1 g of sodium citrate, and 0.5 g of sodium hydrogen citrate sesquihydrate were added to the combined supernatant and shaken for 1 min.

The fat content was removed by treating the extracts with 20 mL of hexane (2 times), vortexing for 1 min, and followed by standing for 5 min to separate the hexane from the extract. For the dispersive SPE clean-up, the bottom layer was transferred into a clean tube that contained 150 mg of C18 sorbent and 900 mg of magnesium sulfate. The cloudy solution was shaken for 1 min and centrifuged at 8000 rpm for 5 min. The supernatant was transferred into a clean tube and washed twice with 5 mL of acetonitrile. The mixture was evaporated to dryness by a rotary evaporator and reconstituted with 1 mL of methanol and filtered through a 0.22 μm nylon syringe filter prior to LC-MS/MS analysis.

### 4.6. Optimization Method 

All experiments were performed by using 100 µg/L concentration for each of AFB1, AFG1, and OTA, and 30 µg/L concentration for each of AFB2, and AFG2. Experimental plans and data interpretations were performed by using MINITAB® Release 17 Statistical Software (State College, PA, USA). The Plackett–Burman design (PBD) was used to screen 11 parameters in 24 runs. Three-level Box–Behnken design (BBD) was carried out in 46 runs in two-block factors. Total chromatographic peak area (TCPA) and mean retention time (MRT) responses were used for response surface analysis. 

### 4.7. Method Performance

#### 4.7.1. Instrument Validation

Some protocols were used to examine the procedure performance (Linearity, IDL, Intra-day precision, and inter-day precision). To study the linearity (R^2^) of calibration curves, standard solutions were prepared at various concentration levels in the mobile phase and in blank food matrices. The extract was injected into the LC-MS/MS system. The instrument detection limit (IDL) is the minimum amount of analyte required for producing a signal which can be statistically distinguished from the background noise level within a specified confidence level. Equation (1) is used in the IDL detection:

IDL = *t* × (RSD/100%) × amount measured (1)

where (*t*) is *t*-Test and RSD is relative standard deviation [48,49].

#### 4.7.2. Detection Method Validation

To evaluate the linearity of method for each of analyte, the matrix-matched calibration curves were in some matrices. The detection limit was estimated as 3:1 signal-to-noise ratio (S/N), while the quantitation limit was estimated at 10:1 signal-to-noise ratio (S/N) for qualifier ion. The recovery was obtained according to Equation (2):

(2) $$\mathrm{RE}\;=\;\frac{C}{B}\;\times \;100$$

where B is the average peak area obtained from the spiking sample after extraction and C is the average peak area obtained from a spiked sample prior to the extraction [50]. 

## Figures and Tables

**Table 1:** Mycotoxins MS data.

Mycotoxin	Molecular Formula	*m/z*	[M + H]^+^	Product Ion (*m/z*)	Collision Energy
AFB1	C_17_H_12_O_6_	312.0634	313.1	241.1	41
				285.2	25
AFB2	C_17_H_14_O_6_	314.0790	315.1	259.1	29
				287.2	29
AFG1	C_17_H_12_O_7_	328.0583	329.1	243.1	29
				215.2	37
AFG2	C_17_H_14_O_7_	330.0740	331.1	313.1	25
				245.0	29
OTA	C_20_H_18_ClNO_6_	403.0823	404.0	221.0	38
				239.0	26

**Table 2:** Liquid chromatography-tandem mass spectrometry (LC-MS/MS) optimization parameters.

Factors	Optimized Univariate Parameter	Optimized Multivariate Parameter	PBD Lower Level	PBD Upper Level	BBD Lower Level	BBD Upper Level
Mobile phase additive	Ammonium formate & formic acid	n.a.	n.a.	n.a.	n.a.	n.a.
Mobile phase pH	3	3	3	7	n.a.	n.a.
Organic solvent (%)	50	60	30	60	40	60
Flow rate (mL/min)	0.15	0.2	0.10	0.20	0.10	0.20
Column temperature (°C)	30	30	25	35	n.a.	n.a.
Injection volume (L)	3	4	2	5	2	4
Sheath gas flow rate (L/min)	11	11	9	12	n.a.	n.a.
Sheath gas temperature (°C)	250	250	150	250	n.a.	n.a.
Gas flow rate (L/min)	12	14	12	18	12	16
Gas temperature (°C)	200	170	150	250	150	250
Nebulizer pressure (psi)	25	25	20	35	n.a.	n.a.
Collision energy (eV)	n.a.	n.a.	25	40	n.a.	n.a.

n.a.: not applicable.

**Table 3:** Box–Behnken Design (BBD) analysis of variance (ANOVA) data.

Source	DF	TCPA		MRT	
		*F*-Value	*p*-Value	*F*-Value	*p*-Value
Model	20	2.53	0.015	133.30	0.000
Blocks	1	4.62	0.042	7.31	0.012
Linear	5	4.18	0.007	547.40	0.000
OS	1	0.91	0.350	1927.53	0.000
FR	1	1.10	0.305	807.80	0.000
IV	1	17.33	0.000	1.60	0.219
GT	1	0.87	0.361	0.06	0.806
GF	1	0.70	0.412	0.01	0.908
Square	5	3.61	0.014	0.82	0.550
OS*OS	1	1.85	0.186	0.44	0.513
FR*FR	1	0.07	0.798	3.02	0.095
IV*IV	1	0.04	0.848	0.19	0.664
GT*GT	1	5.20	0.032	1.18	0.287
GF*GF	1	11.66	0.002	0.02	0.889
2-Way Interaction	10	0.96	0.497	5.10	0.001
OS*FR	1	0.35	0.560	45.54	0.000
OS*IV	1	4.62	0.042	0.95	0.338
OS*GT	1	0.02	0.882	0.43	0.517
OS*GF	1	0.08	0.781	1.26	0.273
FR*IV	1	1.00	0.326	0.75	0.395
FR*GT	1	0.00	0.998	1.72	0.202
FR*GF	1	2.39	0.135	0.00	0.983
IV*GT	1	0.30	0.588	0.12	0.737
IV*GF	1	0.27	0.606	0.01	0.910
GT*GF	1	0.60	0.445	0.21	0.648
Lack-of-Fit	20	0.95	0.593	0.57	0.825

DF: Degree of freedom, FR: flow rate, *F*-Value: Fisher test value, GF: gas flow, GT: gas temperature, IV: injection volume, MRT: Mean retention time, OS: organic solvent, *p*-Value: Probability value, TCPA: Total chromatogram peak area. *: interaction between factors.

**Table 4:** LC-MS/MS validation data.

Mycotoxins	AFB1		AFB2		AFG1		AFG2		OTA	
Range (µg/L)	0.018–50		0.012–15		0.018–50		0.012–15		0.02–50	
	a	b	a	b	a	b	a	b	a	b
R^2^	0.9999	0.9998	0.9994	0.9992	0.9997	0.9995	0.9999	0.9998	0.9998	0.9993
IDL (ng)	1.75	2.35	2.93	3.01	2.60	3.22	3.12	3.61	1.41	2.48
Intra-day Precision (RSD%)	1.32	1.67	0.64	1.84	0.81	1.23	1.17	1.25	2.75	3.67
Inter-day Precision (RSD%)	2.78	2.95	1.63	1.70	0.97	1.80	3.6	3.11	3.3	3.89

AFB1: Aflatoxin B1; AFB2: Aflatoxin B2; AFG1: Aflatoxin G1; AFG2: Aflatoxin G2; OTA: Ochratoxin A; R^2^: regression coefficient; IDL: instrumental detection limit; RSD: relative standard deviation; a: standard in methanol; b: standard spiked in aflatoxin-free peanut extract.

**Table 5:** QuEChERS-LC-MS/MS method concentration range, R^2^, LOD, and LOQ.

Mycotoxins		Apple Juice	Raisin	Wheat Flour	Peanut	Spice Mixture
**AFB1**	Range	1–30	1–30	1–30	1–30	1–30
	R^2^	0.9991	0.9994	0.9993	0.9991	0.9989
	LOD	0.05	0.06	0.05	0.08	0.08
	LOQ	0.08	0.08	0.08	0.13	0.13
**AFB2**	Range	0.3–10	0.3–10	0.3–10	0.3–10	0.3–10
	R^2^	0.9990	0.9989	0.9990	0.9988	0.9987
	LOD	0.06	0.05	0.05	0.08	0.08
	LOQ	0.09	0.09	0.08	0.10	0.10
**AFG1**	Range	1–30	1–30	1–30	1–30	1–30
	R^2^	0.9992	0.9992	0.9991	0.9990	0.9989
	LOD	0.08	0.075	0.08	0.08	0.08
	LOQ	0.13	0.13	0.13	0.13	0.13
**AFG2**	Range	0.3–10	0.3–10	0.3–10	0.3–10	0.3–10
	R^2^	0.9989	0.9986	0.9987	0.9986	0.9984
	LOD	0.06	0.05	0.05	0.08	0.08
	LOQ	0.09	0.09	0.08	0.10	0.10
**OTA**	Range	1–30	1–30	1–30	0.1–30	1–30
	R^2^	0.9991	0.9991	0.9989	0.9968	0.9967
	LOD	0.07	0.07	0.08	0.09	0.1
	LOQ	0.10	0.10	0.20	0.20	0.30

µg/L for liquid samples and µg/kg for non-liquid samples.

**Table 6:** The quick, easy, cheap, effective, rugged, and safe (QuEChERS)-LC-MS/MS method recovery, intra- and inter-day precision.

**Mycotoxins**	**Conc ^a^**		**Apple juice**			**Raisin**					**Wheat Flour**				
			**RE**	**Intra**	**Inter**	**RE**		**Intra**		**Inter**	**RE**		**Intra**		**Inter**
AFB1	5		97.40	1.50	2.12	100.15		0.69		0.82	99.62		3.00		2.49
	10		99.92	1.38	4.01	99.45		1.16		1.28	100.70		0.46		0.43
	30		99.98	0.21	0.23	99.64		3.43		4.86	99.80		0.54		2.30
AFB2	1.5		100.72	0.96	3.38	99.60		1.34		1.10	97.33		1.22		2.48
	3		99.19	1.06	1.33	100.11		2.30		3.57	99.53		1.09		1.16
	10		100.27	1.94	3.27	99.28		1.99		3.31	98.40		1.30		2.57
AFG1	5		96.75	1.17	2.25	98.80		1.61		1.01	99.07		1.17		3.46
	10		100.87	1.75	4.21	99.45		1.07		5.60	100.1		0.65		6.62
	30		99.66	2.84	1.55	98.78		2.68		2.98	101.45		2.82		2.30
AFG2	1.5		96.75	1.18	1.90	98.80		1.60		5.47	88.80		3.01		6.18
	3		97.45	1.10	1.77	99.45		1.06		1.30	96.63		0.65		3.65
	10		99.09	0.70	0.75	97.73		0.60		2.94	97.83		4.04		2.77
OTA	5		96.75	1.17	1.92	90.70		1.61		1.01	94.29		6.37		4.04
	10		99.56	0.48	0.54	97.21		1.03		1.40	100.7		0.66		6.48
	30		98.04	0.55	1.35	98.28		0.12		0.88	97.93		0.55		1.35
		**Conc ^a^**		**Peanut**					**Spice Mixture**						
				**RE**	**Intra**		**Inter**		**RE**			**Intra**		**Inter**	
AFB1		5		98.92	0.61		1.11		93.80			1.65		3.37	
		10		99.88	1.06		1.93		97.81			2.33		1.63	
		30		98.88	0.60		3.29		99.73			0.89		5.55	
AFB2		1.5		92.58	0.80		4.24		90.43			3.16		7.20	
		3		96.31	1.70		2.30		96.81			2.33		**6.81**	
		10		99.93	0.54		2.65		98.54			6.29		4.20	
AFG1		5		95.06	5.48		2.28		99.82			1.17		1.63	
		10		101.67	1.06		10.10		97.81			2.33		8.04	
		30		97.60	1.85		6.27		99.83			0.61		3.34	
AFG2		1.5		88.03	1.17		7.10		84.52			1.18		7.73	
		3		87.78	1.05		6.30		84.22			2.33		9.02	
		10		94.78	0.54		8.55		84.10			3.56		10.28	
OTA		5		91.62	1.17		4.51		85.99			7.25		8.17	
		10		91.97	0.19		4.67		81.94			2.33		9.49	
		30		94.90	0.56		6.36		87.40			0.55		8.71	

Conc: concentration; RE: recovery; Intra: intraday precision; Inter: interday precision. ^a^ µg/L for liquid samples and µg/kg for non-liquid samples.

**Table 7:** Comparison of the developed method with other methods.

Method	Matrix	Mycotoxins	* R^2^	LOQ	* RSD (%)	RE (%)	Ref.
QuEChERS-LC-MS/MS	High oil content (almonds, peanuts, walnuts, hazelnuts, pecan nuts, cashews)	AFG2	>0.9942	1.25	<20	73.66	[36]
		AFG1	>0.9857	1.25	<19	78.00	
		AFB2	>0.9938	1.25	<14	80.00	
		AFB1	>0.9787	1.25	<19	68.33	
		OTA	>0.9939	5.00	<17	76.00	
QuEChERS-LC-MS/MS	High oil content (sesame butter)	AFG2	0.9987	0.21	<6	93.0	[37]
		AFG1	0.9979	0.21	<3	95..0	
		AFB2	0.9983	0.21	<5	97.0	
		AFB1	0.9991	0.21	<5	99.9	
		OTA	0.9987	0.74	-	-	
QuEChERS-LC-MS/MS	High-sugar and high-water content (Grapes and Wines)	AFG2	0.9988	0.18	<18	94.39	[24]
		AFG1	0.9988	0.75	<16	87.95	
		AFB2	0.9993	0.39	<8	94.41	
		AFB1	0.9990	0.75	<11	100.29	
		OTA	0.9998	0.3	<17	96.06	
QuEChERS-LC-MS/MS	Food containing complex components (Different species and medicinal herbs)	AFG2	>0.9996	0.25	<10	76.19	[38]
		AFG1	>0.9947	1.00	<9	82.58	
		AFB2	>0.9968	0.25	<7	87.94	
		AFB1	>0.9933	1.00	<10	84.39	
		OTA	>0.9996	0.5	<16	66.5	
QuEChERS-LC-MS/MS	Different food matrices	AFG2	>0.9984	0.08-0.10	<11	93.06	Present work
		AFG1	>0.9989	0.13	<11	99.11	
		AFB2	>0.9987	0.08-0.10	<7	97.94	
		AFB1	>0.9989	0.08-0.13	<6	99.04	
		OTA	>0.9967	0.10-0.30	<10	93.82	

* “>” signify greater than and “<” signify lesser than; RE: recovery percentage.

**Table 8:** An occurrence of mycotoxins in food samples.

Sample	NS	Concentration (µg/L for Liquid Samples & µg/kg for Non-Liquid Samples)				
		AFB1	AFB2	AFG1	AFG2	OTA
Apple juice	10	n.d.	n.d.	n.d.	n.d.	n.d.
Grape juice	10	n.d.	n.d.	n.d.	n.d.	n.d.
Orange juice	10	n.d.	n.d.	n.d.	n.d.	n.d.
Pomegranate juice	10	n.d.	n.d.	n.d.	n.d.	n.d.
Raisin	10	2.73, 5.67	0.84, 1.33	1.50, 2.50	1.47	n.d.
Dried-fig	10	n.d.	n.d.	n.d.	n.d.	n.d.
Wheat flour	10	1.50, 7.33, 10.12	0.45, 2.70	1.80, 2.61	n.d.	1.2
Barley flour	10	n.d.	n.d.	n.d.	n.d.	n.d.
Non-roasted peanut	10	5.36, 10.23	1.45, 2.22	2.00, 4.35	0.76, 0.82	1.20, 3.53
Roasted pistachio	10	5.30, 5.48, 7.48, 10.15	1.46, 1.60, 3.47	1.90, 2.1, 2.5, 3.31	0.81, 0.90	n.d.
Chili	10	4.90, 5.26, 8.70, 16.93	1.45, 4.69, 8.11	1.76, 1.89, 2.10, 6.96	0.71, 0.96	n.d.
Mixed spice	10	4.70, 7.41, 10.69, 14.36	1.52, 2.26, 3.43, 4.13	1.55, 1.79, 7.74	n.d.	n.d.

n.d.: not detected, NS: Number of samples.

## Supplementary Materials

The following are available online at https://www.mdpi.com/2072-6651/11/4/196/s1, Figure S1: **Figure 1.** TCPA Response surface plot for (**A**) injection volume vs. gas temperature; (**B**) flow rate vs. gas flow; (**C**) gas temperature vs. gas flow; (**D**) injection volume vs. gas flow; (**E**) flow rate vs. gas temperature; (**F**) flow rate vs. injection volume; (**G**) organic solvent vs. gas temperature; (**H**) organic solvent vs. gas temperature; (**I**) organic solvent vs. injection volume; (**J**) organic solvent vs. flow rate; and MRT Response surface plot for (**K**) organic solvent vs. flow rate. Figure S2: Total Ion Chromatogram (TIC) and MRM chromatogram of Mycotoxins Standard from Box-Behnken Design (BBD) Study.
