# Supplementary material for: Detection of Five Mycotoxins in Different Food Matrices in the Malaysian Market by Using Validated Liquid Chromatography Electrospray Ionization Triple Quadrupole Mass Spectrometry
Source: Toxins (Basel). 2019 Mar 31;11(4):196. doi: 10.3390/toxins11040196 (PMC6520768; doi:10.3390/toxins11040196)
Supplement: Supplementary file 1 [file toxins-11-00196-s001.pdf]

Supplementary Materials: Detection of Five Mycotoxins in Different Food Matrices in the Malaysian Market by Using Validated Liquid Chromatography Electrospray Ionization Triple Quadrupole Mass Spectrometry

Ali Mohamed Ali Alsharif, Yeun-Mun Choo and Guan-Huat Tan

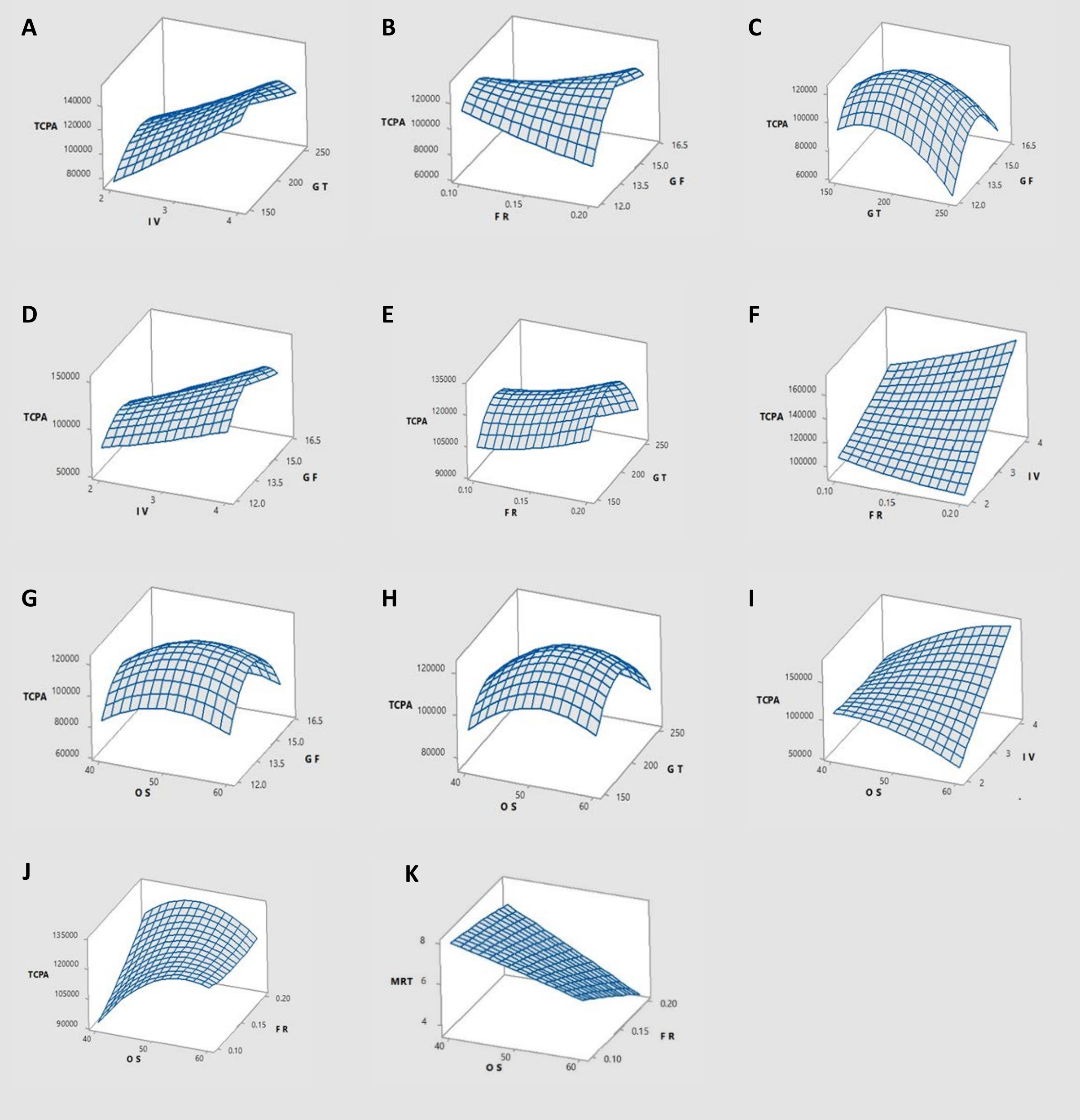

Figure 1. TCPA Response surface plot for (**A**) injection volume vs. gas temperature; (**B**) flow rate vs. gas flow; (**C**) gas temperature vs. gas flow; (**D**) injection volume vs. gas flow; (**E**) flow rate vs. gas temperature; (**F**) flow rate vs. injection volume; (**G**) organic solvent vs. gas temperature; (**H**) organic solvent vs. gas temperature; (**I**) organic solvent vs. injection volume; (**J**) organic solvent vs. flow rate; and MRT Response surface plot for (**K**) organic solvent vs. flow rate.

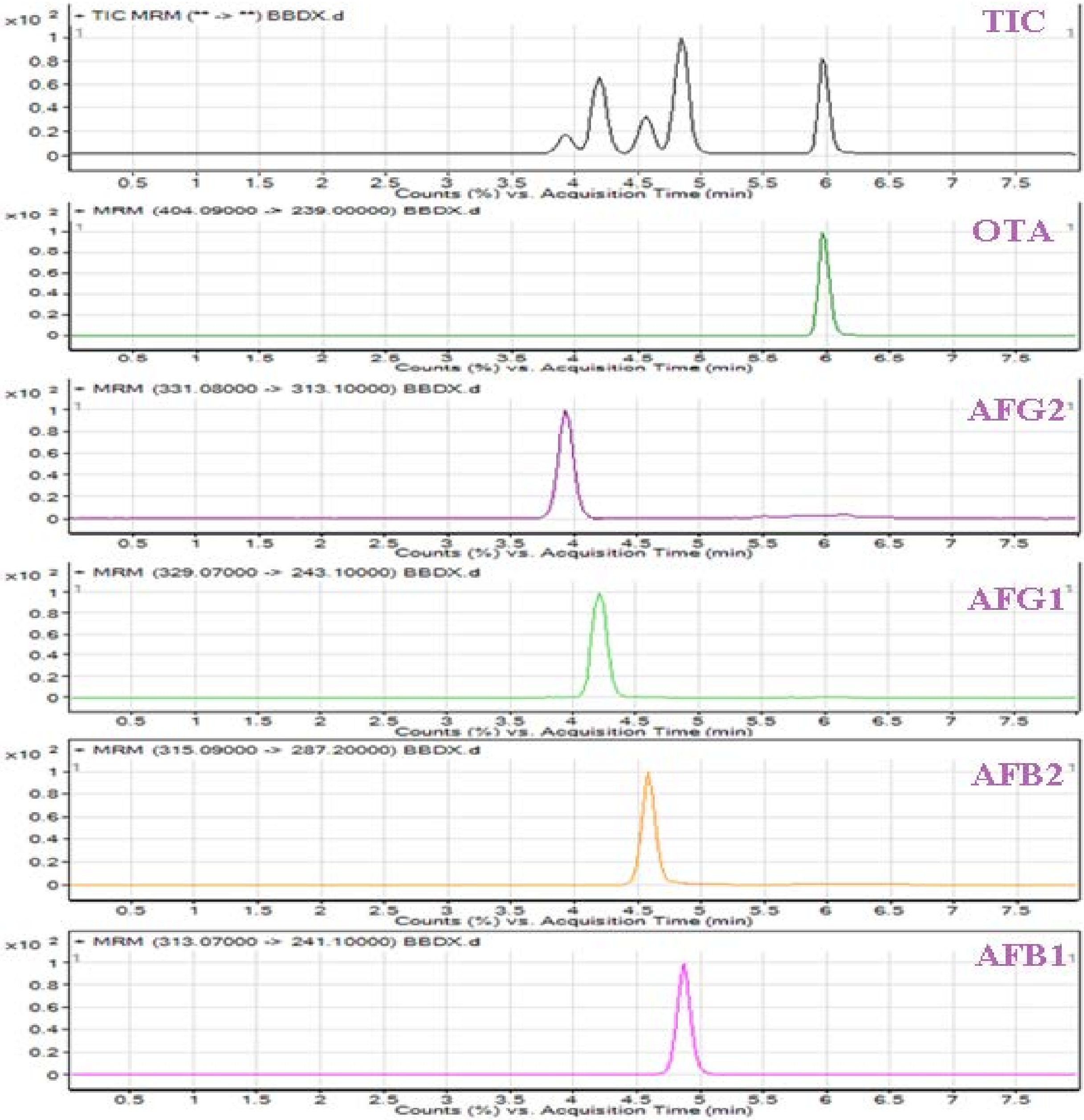

Figure 2. Total Ion Chromatogram (TIC) and MRM chromatogram of Mycotoxins Standard from Box-Behnken Design (BBD) Study.
